# Hormone Replacement Versus Natural Cycle Protocols of Endometrial Preparation for Frozen Embryo Transfer

**DOI:** 10.3389/fendo.2020.546532

**Published:** 2020-09-30

**Authors:** Ye Pan, Bo Li, Ze Wang, Ying Wang, Xiaoshu Gong, Wenqing Zhou, Yuhua Shi

**Affiliations:** ^1^Center for Reproductive Medicine, Cheeloo College of Medicine, Shandong University, Jinan, China; ^2^Key Laboratory of Reproductive Endocrinology of Ministry of Education, Shandong University, Jinan, China; ^3^Shandong Key Laboratory of Reproductive Medicine, Jinan, China; ^4^Shandong Provincial Clinical Research Center for Reproductive Health, Jinan, China; ^5^National Research Center for Assisted Reproductive Technology and Reproductive Genetics, Shandong University, Jinan, China; ^6^Qingdao Women and Children’s Hospital, Qingdao, China

**Keywords:** caesarean section, frozen embryo transfer, hormone replacement therapy cycle, natural cycle, implantation rate

## Abstract

**Research question:**

Endometrial preparation is one of the most important steps for ensuring frozen embryo transfer success. However, there is no clear evidence that identifies an optimal endometrial preparation protocol for frozen embryo transfer. In addition, in studies that assessed which were the optimal endometrial preparation protocols, few analyzed the stage and the number of embryos. This study compared the pregnancy outcomes and perinatal obstetric complications of patients who were transferred two cleavage-stage (day 2 or day 3) frozen embryos with the natural cycle and those with the hormone replacement therapy cycle.

**Design:**

This study was a secondary analysis of data from a multicentre randomized controlled trial designed to compare the pregnancy and perinatal outcomes after frozen versus fresh embryo transfer. In this study, a total of 908 patients who were transferred two cleavage-stage (day 2 or day 3) embryos in the original trial were analyzed. Pregnancy outcomes and perinatal obstetric complications after the natural cycle and the hormone replacement therapy cycle were compared.

**Result:**

We found the endometrium in the natural group was significantly thicker than the hormone replacement therapy cycle group (*p*<0.01). The implantation rate (42.6% vs 37.3% *p*=0.049) showed a significant difference between the natural cycle group and the hormone replacement therapy cycle group. Compared to the natural cycle group, the hormone replacement therapy cycle group was associated with an increased risk of caesarean section (72.3% vs 84.5, *p*=0.009).

**Conclusion:**

The natural cycle protocol yielded thicker endometria, a higher implantation rate and a lower risk of caesarean section than the hormone replacement therapy protocol in the transfer of two cleavage-stage frozen embryos. The natural cycle protocol was the better endometrial preparation protocol for frozen embryo transfer.

## Introduction

In 1983, the first successful pregnant of frozen embryo transfer (FET) was reported in humans, and FET technology has been widely applied in the clinic ever since (1). FET use can reduce the risk of ovarian hyperstimulation syndrome (OHSS) and decrease the incidence of multiple pregnancies (2, 3). In recent years, the development of cryopreservation technology has made the frozen-thawed embryo process safer. Many studies have verified that the pregnancy outcome of FET was no worse than outcomes following fresh embryo transfer; even in women with a high ovarian response, the pregnancy outcomes for FET were better than the fresh embryo transfer (4–6). Furthermore, because of avoiding the influence of supra-physiologic hormonal levels in controlled ovarian stimulation, which enables the process of assisted reproductive technology to be safer and improves pregnancy outcome, FET (even the freeze-all strategy) has been used with increasing frequency (7, 8). However, some studies also demonstrated that FET has adverse effects on obstetric outcomes, such as hypertension during pregnancy, large for gestational age and macrosomia (9, 10). Recently, some reports indicated that the protocols of endometrial preparation may be relevant for pregnancy and obstetric outcomes after FET (11, 12). Moreover, endometrial preparation is one of the most important steps for ensuring FET success. Therefore, identifying whether the protocols used for endometrial preparation influence the outcomes of FET is a primary research interest at the present time.

Many regimens of endometrial preparation exist; they are mainly the natural cycle and the hormone replacement therapy (HRT) cycle (13). In the natural FET cycle, endometrial maturation relies on endogenous oestradiol (E2) and progesterone, which are created by the growth of a dominant follicle and are used to stimulate the growth of the endometrium. This method avoids the preparation of the endometrium by treatment with exogenous hormone, and it is simpler and more physiological than other methods. Hence, the natural cycle has always been the first choice of many clinicians. In the HRT cycle, supplementing exogenous oestrogen is used to stimulate the growth of the endometrium and inhibit follicular growth. The HRT cycle is more flexible and convenient, is good for arranging transplantation, and has a lower cancellation rate compared with the natural cycle, which is the reason that some clinicians have preferred to choose the HRT cycle. However, each method has its own shortcomings. The disadvantages of the natural cycle are the intensive monitoring of ovulation and the unsuitability of the method for patients with ovulatory dysfunction (14). The main drawbacks of the HRT cycle method are the potential adverse risks caused by exogenous oestrogen supplementation and the higher cost (15). Despite many studies investigating these methods, there is no consensus on the most effective method for clinically preparing the endometrium. Moreover, most of the currently available studies on endometrial preparation protocols did not distinguish cleavage stage or blastocyst stage and did not determine the number of transferred embryos.

Recently, a large multicentre randomized controlled trial (RCT) in ovulatory women comparing the pregnancy outcome and obstetric perinatal complications after frozen versus fresh embryo transfer showed that pregnancy outcomes and obstetric perinatal complications were not significantly different between the frozen and the fresh embryo transfer groups (5). Therefore, we performed a secondary analysis among the patients who transferred two cleavage-stage (day 2 or day 3) frozen embryos in the RCT, and we compared pregnancy and perinatal period outcomes of two different regimens of preparation the endometrium. The purpose of the study was to determine the optimized protocols for preparing the endometrium among patients who were transferred two cleavage-stage frozen embryos.

## Materials and Methods

### Study Procedures

The present study was a secondary analysis that analyzed FET patients’ data from an original RCT to compare the pregnancy and perinatal period outcomes of two different protocols of endometrial preparation (natural cycle and HRT) among ovulatory women. The original RCT was designed to compare the pregnancy and perinatal outcomes after frozen versus fresh embryo transfer. It was conducted during March 2015 and May 2017 including 20 reproductive medical centers in China, the study enrolled 2,157 infertile patients who had no ovulation disorder. The RCT was approved by Ethics Committee at Center for Reproductive Medicine, Shandong Provincial Hospital Affiliated to Shandong University, and with the Helsinki Declaration of 1975. Every enrolled couple signed informed consent forms. This study analyzed 908 patients who transferred two cleavage-stage (day 2 or day 3) frozen embryos in that RCT. We excluded patients who transferred fresh embryos, blastocyst embryos, and one embryo and patients who had missing data. The designs and protocols of the trial have been previously reported, and the details are available (5). Briefly, the inclusion criteria are shown below: (1) Women whose age was >20 and ≤ 35 years. (2) Women who were regularly menstruating with a cycle length of 21~35 days. (3) Women who were undergoing their first *in vitro* fertilization (IVF)/intracytoplasmic sperm injection (ICSI) cycle. (3) Women who had more than 5 oocytes retrieved. The exclusion criteria were as follows: (1) Women who were diagnosed with uterine anatomic abnormalities, either inborn or acquired. (2) Women who had one ovary removed. (3) Women who were polycystic ovary syndrome patients. (4) Women who needed preimplantation genetic testing. (5) Women who suffered from recurrent miscarriages. (6) Women with medical conditions who were interdicted assisted reproductive technology (ART)/pregnancy. A gonadotropin-releasing hormone antagonist cycle was adopted for ovarian stimulation in this trial.

In this study, all participants were divided into the natural cycle group and the HRT cycle group according to the endometrial preparation protocols. The protocols for the endometrial preparation were determined by the local investigators in the primary RCT. The natural cycle was regarded as the optimal choice. If the natural cycle failed to result in good preparation, an artificial cycle was used in the next menstrual cycle. In the natural cycle group, the participants were monitored for ovulation using ultrasound. And the specific protocol for monitoring ovulation was determined by the local investigators. Luteal phase support with oral dydrogesterone 10 mg twice a day was started from the day of ovulation. According to day 2 or day 3 frozen embryos, two cleavage-stage frozen embryos were transferred after two days or three days, respectively, of ovulation. If the participant became pregnant, luteal phase support was continued until 10 weeks of gestation. For the HRT cycle group, oral oestradiol valerate was given daily at a dose of 4**–**8 mg started on the 1**–**3 day of the period. When the endometrial thickness reached 7 mm or more, twice daily oral dydrogesterone (10 mg) and vaginal progesterone gel (90 mg/day) were added. According to day 2 or day 3 frozen embryos, two cleavage-stage frozen embryos were transferred after two days or three days, respectively, of using progesterone. If the participants were pregnant, oral oestradiol valerate was gradually diminished, vaginal progesterone was continued until clinical pregnancy, and oral dydrogesterone was continued until 10 weeks gestation. The follow-up of all participants was continued until 6 weeks after birth.

### Outcomes

The pregnancy outcomes of this study included biochemical pregnancy, clinical pregnancy, implantation rate, ongoing pregnancy, live birth, and pregnancy loss. Biochemical pregnancy was defined as β-HCG (human chorionic gonadotropin) serum level of at least 10 IU per litre at 15 days after embryo transfer. Clinical pregnancy was defined by the presence of intrauterine gestation sacs at 35 days after transfer. The implantation rate was defined as the number of gestational sacs (determined by sonogram) divided by the number of frozen embryos transferred. Ongoing pregnancy was defined as a viable foetal heartbeat at 11 weeks of gestation. Live birth was defined as delivery of any neonate after 28 weeks of gestation. Pregnancy loss was defined as all abortions occurring throughout pregnancy.

The outcome of obstetric complications included gestational diabetes mellitus, hypertension during pregnancy (HDP), premature membrane rupture, caesarean section, low birth weight infant, macrosomia, small for gestational age (SGA), large for gestational age (LGA) and malformation. Hypertension during pregnancy in this study includes preeclampsia and gestational hypertension. A low birth weight infant was defined as an infant weighing less than 2,500 g. Macrosomia was defined as birthweight greater than 4,000 g. SGA was defined as birthweight <10th percentile of reference standard birthweight for gestational age. LGA was defined as birthweight >90th percentile of reference standard birthweight for gestational age. The standard birthweight was based on Chinese populations and were adjusted for sex and gestational age (16).

### Statistical Analysis

Data of continuous variables were expressed as the mean ± standard deviation (SD) for normally distributed variables or as median (25th**–**75th percentile) for non-normal distributed variables. Data of categorical variables were expressed as a percentage. Means of two continuous normally distributed variables were compared by independent samples Student’s t-tests. Mann-Whitney U tests were used to compare two groups of variables not normally distributed. Chi-squared tests or Fisher’s exact tests were used to compare the distributions of categorical variables between two groups. Statistical analysis was performed using SPSS version 22 (SPSS Inc., Chicago, IL, USA). A value of *P* <0.05 was considered statistically significant.

## Results

A total of 908 frozen embryo transfer cycles were analysed; 683 were natural cycles, and 225 were HRT cycles. The baseline characteristics are shown in **Table 1**. Patients in the natural cycle group underwent a longer duration of infertility and lower body mass index (BMI) than patients in the HRT cycle group. After endometrial preparation, the endometrium in the natural group was significantly thicker than it was in the HRT cycle group (*p*<0.01). In addition, there were no significant differences between the groups in other baseline characteristics.

**Table 1:** Baseline characteristics.

Characteristics	Total n = 908	Natural cycle n = 683(75.2)	HRT cycle n = 225(25.8)	*P* value
Age(years)	28.41 ± 3.00	28.49 ± 2.98	28.18 ± 3.07	0.186
Duration of infertility (years)	3[2**–**4.5]	3[2**–**5]	3[1.5**–**4]	0.005
Indication for assisted reproductive technology (%)				0.217
Male factor	558(61.5)	412(60.3)	146(64.9)	
Tubal factor	236(26.0)	178(26.1)	58(25.8)	
Combined factors	114(12.5)	93(13.6)	21(9.3)	
Body-mass index (kg/m²)	21.98 ± 2.98	21.83 ± 2.90	22.44 ± 3.18	0.008
Body-mass index (%)				0.092
<18.5	87(9.6)	67/683(9.8)	20/225(8.9)	
18.5**–**23.9	628(69.2)	484/683(70.9)	144/225(64.0)	
24**–**27.9	154(17.0)	104/683(15.2)	50/225(22.2)	
≥28	39(4.3)	28/683(4.1)	11/225(24.9)	
Follicle-stimulating hormone(IU/L)	6.58 ± 1.64	6.63 ± 1.63	6.43 ± 1.67	0.117
Luteinizing hormone(IU/L)	4.97 ± 1.92	4.98 ± 1.88	4.96 ± 2.03	0.924
Fertilisation method, number/total number (%)				0.057
In vitro fertilization	591(65.1)	443/683(64.9)	148/225(65.8)	
Intracytoplasmic sperm injection	275(30.3)	202/683(29.6)	73/225(32.4)	
Other	42(4.6)	38/683(5.6)	4/225(1.8)	
Timing of embryo transfer-no.(%)				0.917
Day2	21(2.3)	16/683(2.3)	5/225(2.2)	
Day3	887(97.7)	667/683(97.7)	220/225(97.8)	
Number of oocytes retrieved, number	12[9**–**16]	12[9**–**16]	13[10**–**17]	0.402
Thickness of the endometrium, cm	1[0.85**–**1.10]	1[0.9**–**1.1]	0.9[0.83**–**0.9]	0.000

Normally distributed variables were presented as means ± SD and Non-normally distributed variables were presented as median [25th**–**75th percentile].

Categorical variables were presented as frequencies and percentages.

**Figure 1:**
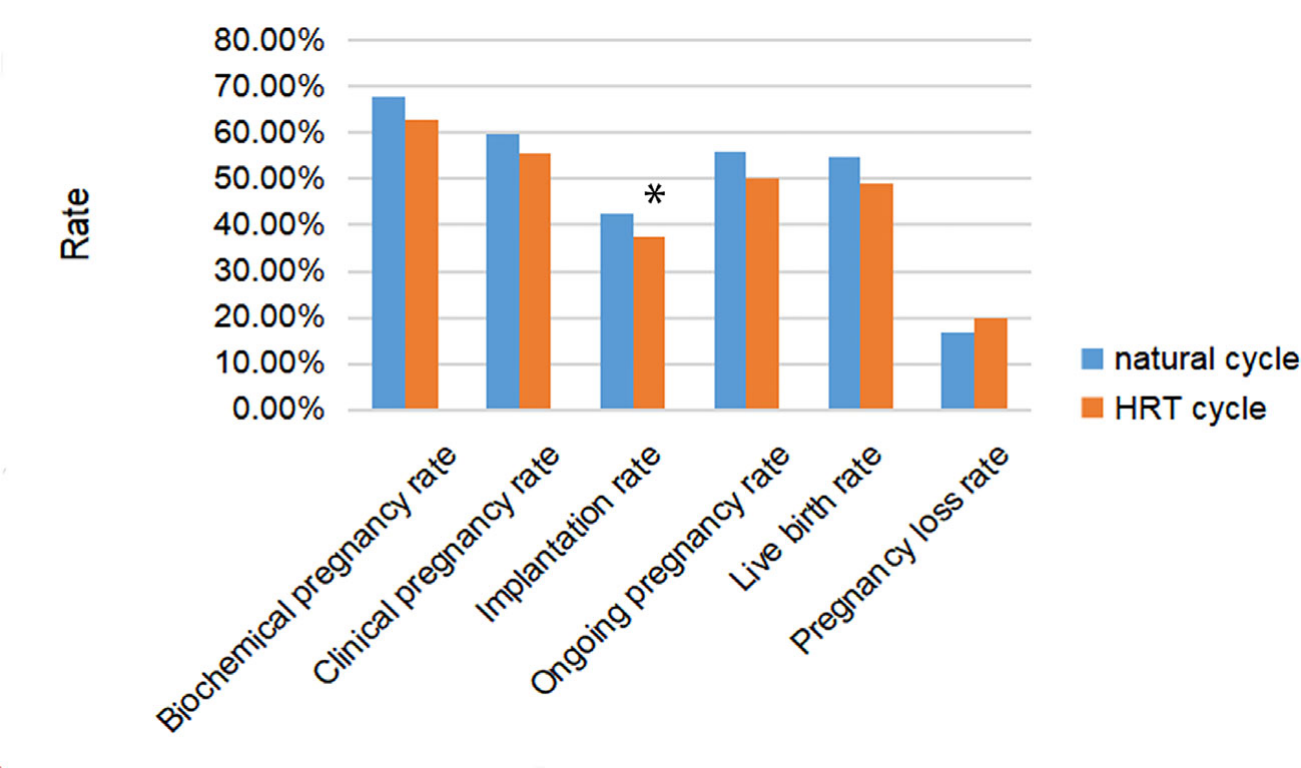
Rates of pregnancy, implantation, live birth and pregnancy loss after frozen embryo transfer (FET) among women with natural cycle or hormone replacement therapy (HRT) cycle for endometrial preparing. * represented the difference among three groups was statistically significant.

Pregnancy outcomes are shown in **Table 2**. In all cycles, the live birth rate was 53.4% (485/908), producing a total of 666 newborns, including 181 pairs of twins. All pregnancy outcomes of the natural cycle tended to be better than the HRT cycle, as shown in **Figure 1**. However, only the implantation rate (42.6% vs 37.3% *p*=0.049) showed a significant difference between the natural cycle group and the HRT cycle group. The comparison between the two groups did not reveal significant differences in biochemical pregnancy, clinical pregnancy, ongoing pregnancy, live birth, or pregnancy loss.

**Table 2:** Pregnancy outcomes after frozen embryo transfer (FET) treatment.

Variables	Total	Natural cycle	HRT cycle	*P* value
Biochemical pregnancy, number/total number (%)	604/908(66.5)	463/683(67.8)	141/225(62.7)	0.158
Clinical pregnancy, number/total number (%)	533/908(58.7)	408/683(59.7)	125/225(55.6)	0.269
Implantation, number/total number (%)	750/1816(41.3)	582/1366(42.6)	168/450(37.3)	0.049
Ongoing pregnancy, number/total number (%)	494/908(54.4)	381/683(55.8)	113/225(50.2)	0.146
Pregnancy loss (total), number/total number (%)	105/604(17.4)	77/463(16.6)	28/141(19.9)	0.376
Live birth, number/total number (%)	485/908(53.4)	375/683(54.9)	110/225(48.9)	0.117
Singleton live birth	304/485(62.7)	229/375(61.1)	75/110(68.2)	
Twins live birth	181/485(37.3)	146/375(38.7)	35/110(31.8)	

The obstetric and perinatal outcomes are listed in **Table 3**. Gestational diabetes mellitus, hypertension during pregnancy and premature rupture of the membrane were not significantly different between the two groups. There was a significant difference in caesarean section between the natural cycle group and the HRT cycle group, respectively (72.3% vs 84.5% *p*=0.009); the HRT cycle group was associated with an increased risk of caesarean section compared to the natural cycle group. The neonatal of gestational age, newborn sex and malformation rates were comparable between the two groups. No significant difference was found in newborn weight, low birth weight infants, macrosomia, SGA, and LGA between the two groups.

**Table 3:** Obstetric and perinatal outcomes.

Variables	Total	Natural cycle	HRT cycle	*P* value
Gestational diabetes mellitus, number/total number (%)	17/533(3.2)	16/408(4.2)	1/125(0.8)	0.140
Hypertension during pregnancy, number/total number (%)	30/533(5.6)	24/408(5.9)	6/125(4.8)	0.646
Premature rupture of the membrane, number/total number (%)	19/533(3.6)	16/408(3.9)	3/125(2.4)	0.598
Caesarean section, number/total number (%)	364/485(75.1)	271/375(72.3)	93/110(84.5)	0.009
Gestational age, weeks	38[37-39]	38[37-39]	38[37-40]	0.666
Gestational age category, number/total number (%)				0.312
<37 weeks	83/485(17.1)	63/375(16.8)	20/110(18.2)	
37-41 weeks	372/485(76.7)	292/375(77.9)	80/110(72.7)	
≥41 weeks	30/485(6.2)	20/375(5.3)	10/110(9.1)	
Sex of the neonate, number/total number (%)				0.251
Male	345/666(51.8)	276/521(53.0)	69/145(47.6)	
Female	321/666(48.2)	245/521(47.0)	76/145(52.4)	
Birth weight, g	3008.54 ± 571.069	2996.77 ± 574.27	3051.11 ± 559.22	0.313
Low birth weight infant, number/total number (%)	113/665(17)	89/521(17.1)	24/144(16.7)	0.906
Macrosomia, number/total number (%)	35/665(5.3)	27/521(5.2)	8/144(5.6)	0.859
Small for gestational age, number/total number (%)	93/665(14)	75/521(14.4)	18/144(12.5)	0.562
Large for gestational age, number/total number (%)	63/665(9.5)	48/521(9.2)	15/144(10.4)	0.662
Malformation, number/total number (%)	19/666(2.9)	16/521(3.1)	3/145(2.1)	0.720

Birthweight of one newborn baby who was one of a twin was missing.

## Discussion

This study showed that the natural cycle yielded a thicker endometrium, a higher implantation rate and a lower risk of caesarean section compared with the HRT cycle in two frozen cleavage-stage embryo transfers. There was no significant difference in the live birth rate or other perinatal obstetric complications between the two protocols.

Endometrial preparation is one of the most important steps to ensure the success of FET. The adequate thickness of the endometrium before FET and successful implantation after FET are important to achieving final successful live birth (17). Ashrafi et al. (18) considered endometrial thickness to be one of the most important factors affecting implantation rate and clinical pregnancy rate. Bu et al. (19) concluded that in cleavage-stage frozen embryo transfer, endometrial thickness significantly affected IVF outcomes. In this study, the natural cycle yielded thicker endometria than the HRT cycle. This result might suggest that using the natural cycle could be better than the HRT cycle. However, a further study is needed to confirm the superiority of the natural cycle in terms of the thickness of the endometrium because this study was not randomized and HRT cycles were used in some participants after a natural cycle failed.

Previous studies demonstrated that high serum E2 levels could damage the endometrium and shorten the available implantation window and inhibit embryo implantation in the endometrium (20, 21). Exogenous E2 was used in HRT cycles, and the E2 levels of the endometrium exposed in the HRT cycles were higher than they were in the natural cycle (22). This difference indicated that the implantation rate of the frozen embryos was influenced in the HRT cycle. In this study, we found that the implantation rate of the natural cycle was better than that of the HRT cycle. All pregnancy outcomes of the natural cycle tended to be better than those of the HRT cycle. However, except for the implantation rate, there were no significant differences in the clinical pregnancy rate and live birth rate between the natural cycle and the HRT cycle. The result of this study was similar to previous studies. Kawamura et al. (17) and Hancke et al. (23) found that, compared with the HRT cycle, the natural cycle demonstrated a trend towards higher live birth rates, but the difference in the two groups was not statistically significant. Levron et al. (14) performed a retrospective analysis involving 1,235 FET cycles of cleavage stage embryos and found that the natural cycle yielded a higher endometrial thickness, implantation rate, and clinical pregnancy rate than the HRT cycle did. Moreover, most studies available at present document that there is a lack of evidence to support one method of endometrial preparation being superior to the others (24). A recent Cochrane analysis also found that no method is better (25). In contrast, some published studies present different results. Saito et al. (12) found that the pregnancy rate and the live birth rate in HRT cycles were significantly lower than they were in natural cycles. Zheng et al. (26) documented that patients undergoing an HRT cycle obtained a higher implantation rate and clinical pregnancy rate than patients undergoing the natural cycle. Which protocol of endometrial preparation could obtain better pregnancy outcomes is subject to debate. Further studies need to be performed in the future.

In our study, of the perinatal obstetric complications from the transfer of in two cleavage-stage frozen embryos, the caesarean section risk rate was significantly different between the natural cycle and the HRT cycle. This result was similar to a previous finding performed by Saito K et al. (27), who considered that HRT cycles increased the risk of caesarean section compared with natural cycles. However, they also found that HRT cycles increased the risk of post-term delivery compared to what was observed with the natural cycles; this result was not found in our study. Participants in our study did not have polycystic ovary syndrome and were not at a high risk for adverse obstetric outcomes, which suggested that the protocol of endometrial preparation using the HRT cycle might be responsible for the increased risk of caesarean section. The potential mechanism underlying the increased risk of caesarean section in the HRT cycles might include disrupted oestrogen and progesterone in the first trimester. In the HRT cycle, the high level of serum oestrogens in early pregnancy might lead to adverse placental angiogenesis, trophoblast development, and invasion in the HRT cycle, which might result in placenta-related complications such as HDP and placenta accrete (28, 29). Placental insufficiency, HDP, post-term delivery, and macrosomia were the frequent reasons for caesarean section. In our study, no significant difference could be found regarding HDP, post-term delivery and macrosomia between the two protocols. However, some studies have indicated different conclusions. Ginström Ernstad et al. (30) found that the increased risks of HDP, postpartum haemorrhage, post-term delivery, and macrosomia were detected in HRT cycles. Saito et al. (12) reported that the HRT cycle was significantly associated with increased risks of HDP, post-term delivery, caesarean section, placenta accrete, macrosomia, and LGA and decreased risks of gestational diabetes mellitus. The conflicting results might be due to the small sample size of the HRT cycle in our study compared with those studies. In addition, recent studies have indicated an association between BMI and cesarean delivery after IVF. However, these studies only found an increased risk for cesarean delivery in overweight and obese women undergoing ART (31–33). In this study, participants in the natural cycle group had a lower BMI than did those in the HRT cycle group. Nevertheless, no significant difference in the categorized BMI values was found between the two groups. Thus, we think that the difference in the frequency of cesarean section delivery between the natural-cycle and HRT cycle groups was unlikely to be due to the difference in the baseline BMI values.

In this study, we found a thicker endometrium, a higher implantation rate and a lower risk of caesarean section in the natural cycle compared with the HRT cycle. Our study adds some evidence for the natural cycle being the first choice in ovulatory women with two cleavage-stage frozen embryos transfer. Although there were no statistically significant differences in the live birth rate between the groups, all pregnancy outcomes of the natural cycle tended to be better than those from the HRT cycle; the implantation rate was better in the natural cycle, and the risk of caesarean section in the natural cycle was significantly lower than it was in the HRT cycle. Besides, the natural cycle is simple, is more physiologically natural and does not require additional medications, which reduces the burden of medication for patients. The HRT cycle requires additional medications and a condition of high E2 levels, and those factors might influence the condition of the endometrium and the placental development; therefore, those factors may be unfavorable for embryo implantation and increase the risk of obstetrical complications (12). In the future, large randomized controlled trials need to be performed to determine the most effective method of endometrial preparation in the clinic. Further studies need to clarify the potential mechanism underlying the HRT cycle affecting pregnancy outcome and obstetrical complications.

The limitation of this study is the small sample size of the HRT cycle group. The natural cycle is the first choice. An artificial cycle was used for endometrial preparation in patients who did not achieve adequate preparation with the natural cycle and in some first-time IVF patients. We did not record who were switched from first attempt natural cycle to HRT. Besides, all participants are all below 35 years. This results in selection bias. In this study, the criteria of the protocol excluded most of the gynecologic disorders that could affect endometrial receptivity. So, these results cannot cover the patients with insufficient endometrial receptivity. This study cannot indicate that the different protocols of endometrial preparation impact the pregnant outcome in insufficient endometrial receptivity. Most patients transferred two D3 frozen embryos and some patients have transferred two D2 frozen embryos that are confounding factors. Hence, these observations must be interpreted cautiously. The advantage of this study is that the original data come from our previous RCT; the baseline data, pregnancy outcomes, and follow-up outcomes are well documented and reliable. In addition, this study analyses the stage and number of embryos.

## Conclusions

In conclusion, the natural cycle yielded thicker endometria, a higher implantation rate and a lower risk of caesarean section compared to the HRT cycle in two cleavage-stage frozen embryo transfers. There was no significant difference in live birth rate or other maternal or neonatal complications between the two protocols. This result adds some evidence supporting the natural cycle being the first choice in ovulatory women with two cleavage-stage frozen embryo transfers. Further studies with larger numbers of patients and prospective randomized clinical trials are needed to confirm the most effective method of endometrial preparation in the clinic.

## Data Availability Statement

The raw data supporting the conclusions of this article will be made available by the authors, without undue reservation.

## Ethics Statement

The studies involving human participants were reviewed and approved by Ethics Committee at Center for Reproductive Medicine, Shandong Provincial Hospital Affiliated to Shandong University. The patients/participants provided their written informed consent to participate in this study. Written informed consent was obtained from the individual(s) for the publication of any potentially identifiable images or data included in this article.

## Author Contributions

YS designed the trial and were in charge of the trial conduct. YP and BL designed the study. YS and ZW acquired the data. BL, YP, and XG performed the statistical analyses. YP, BL, WZ, XG, and YW interpreted the data. YP wrote the first draft of the report with inputs from YW, WZ, and YS provided comments, participated in additional discussions, and revised the paper. All authors contributed to the article and approved the submitted version.

## Funding

This work was supported by the National Key R&D Program of China (grant numbers 2018YFC1003202, 2017YFC1001004) and the Taishan scholar project special funds (grant numbers No. ts201712103).

## Conflict of Interest

The authors declare that the research was conducted in the absence of any commercial or financial relationships that could be construed as a potential conflict of interest.
